# Value of sputum Gram stain, sputum culture, and bronchoalveolar lavage fluid Gram stain in predicting single bacterial pathogen among children with community-acquired pneumonia

**DOI:** 10.1186/s12890-022-02234-1

**Published:** 2022-11-19

**Authors:** Ruimu Zhang, Yue Wu, Guangcheng Deng, Jikui Deng

**Affiliations:** 1 Department of Infectious Diseases, Shenzhen Children’s Hospital, Shenzhen, 518038 China; 2 Department of Clinical Pharmacy, Shenzhen Children’s Hospital, Shenzhen, 518038 China; 3 Faculty of Medicine, Nursing and Health Science, Monash University, Melbourne, Australia

**Keywords:** Gram stain, Sputum, Bronchoalveolar lavage fluid, Pneumonia, Children

## Abstract

**Background:**

Currently, the microbial etiology of community-acquired pneumonia in children remains challenging. While Gram stain and sputum culture are commonly used to detect bacterial pathogens, it is unclear whether these approaches can predict single pathogen from bronchoalveolar lavage fluid (BALF) culture.

**Methods:**

A retrospective study involving 287 children hospitalized for pneumonia was conducted. Sputum specimens were collected on admission; and BALF specimens were collected within 24 h after admission. Taking BALF culture as the reference standard, the sensitivity and specificity of Sputum Gram stain (SGS), sputum culture, and BALF Gram stain (BGS) were calculated. The agreement between these approaches and BALF culture was compared using kappa statistics.

**Results:**

For SGS, the specificity was 23%. The overall sensitivity was 70%, including 87% for Gram-positive (G+) cocci, 56% for Gram-negative (G-) cocci, and 50% for G-bacilli. For sputum culture, the specificity was 70%. The overall sensitivity was 64%, including 71% for *Streptococcus pneumoniae*, 71% for *Moraxella catarrhalis*, and 64% for *Haemophilus influenzae*. For BGS, the specificity was 71%. The overall sensitivity was 60%, including 77% for G+cocci, 38% for G-cocci, and 44% for G-bacilli. While SGS had poor agreement with BALF culture, both sputum culture and BGS had moderate agreement with BALF culture.

**Conclusions:**

Both sputum culture and BGS are helpful in predicting single bacterial pathogen from BALF culture among children with community-acquired pneumonia. Sputum cultures and BGS can provide early clues for BALF pathogen when BALF culture results are pending or bronchoscopy is not performed.

## Background

Pneumonia is the single largest infectious cause of death in children worldwide. It killed 740 180 children under the age of 5 in 2019, accounting for 14% of all deaths of children under five years old and 22% of all deaths in children aged 1 to 5 years [1]. Bacterial infection is one of the most common causes of pneumonia. Accurate and timely detection of the causative bacterium is critical for both the targeted antimicrobial therapy and reduction of antimicrobial resistance [2].

Currently, the microbial etiology of community-acquired pneumonia in children remains challenging [3]. Although various biological specimens and diverse methods have been used to identify organisms [4], etiologic diagnoses were not established in approximately half of community-acquired pneumonia cases [5].

Apart from lung tissue or aspirate, bronchoalveolar lavage fluid (BALF) is the most ideal specimen for detecting the causative pathogen [6]. However, bronchoscopy is unavailable or unnecessary in most children with pneumonia. Furthermore, it usually requires more than 2 days to isolate pathogens from BALF cultures and perform antibiotic susceptibility tests, which may delay the targeted antibacterial therapy.

Sputum Gram stain (SGS) is a quick, convenient and inexpensive approach commonly used to detect bacterial pathogens in pneumonia. Previous studies assessing the diagnostic accuracy of SGS reported heterogeneous results with limited conclusions, as the reference standards were various [7–9]. Compared with SGS, sputum culture requires longer time and BALF Gram stain (BGS) is less widely performed. Though they are also used for pathogen detection, their value in detecting causative pathogen is also unclear. A simple comparison of sputum culture and BALF culture was made in some studies, but the agreement between these two methods was seldom analyzed [10, 11]. Few studies assessing the diagnostic accuracy of BGS used BALF culture as the reference standard. Thus, the concordance between these diagnostic approaches and BALF culture is unclear.

In this study, we assessed the value of SGS, sputum culture, and BGS with single bacterial type in predicting single bacterial pathogen from BALF culture.

## Methods

### Subjects

From January 1, 2015 to December 31, 2021, children hospitalized for pneumonia with BALF collected within 24 h after admission were included in the study from Shenzhen Children’s Hospital, Shenzhen, China.

The inclusion criteria were as follows: aged < 18 years old; radiological evidence of pneumonia before admission or within 24 h after admission; BALF culture performed within 24 h after admission. Patients were excluded if they met one of the following exclusion criteria: diagnosis of pulmonary tuberculosis, fungal infection, or parasite infection; presence of different bacteria where a predominant type was not identified (mixed bacteria) in BALF culture; mixed bacteria in SGS, sputum culture, and BGS; lack of sputum culture and poor quality of Gram stain; none of the SGS, BGS, or sputum culture was performed.

### Specimen collection

Expectorated sputum samples were collected on admission in children who could expectorate. Nasotracheal suctioned sputum samples were collected by the attending nurse in children who could not expectorate. BALF specimens were collected directly after electronic bronchoscopy, which was performed within 24 h after admission. All the samples were then sent to the lab immediately and tested by Gram stain and bacterial culture. The laboratory staff were blinded to the final diagnosis.

### Gram stain and bacterial culture

Gram stain was performed and interpreted by senior laboratory staff. Samples were considered of good quality if they contain ≥ 25 polymorphonuclear cells and < 10 squamous epithelial cells per low-power field. Otherwise, samples were considered poor quality. In good quality samples, > 10 microorganisms of same morphotype at oil immersion field were considered as meaningful.

For bacterial culture, a quality evaluation by smear microscopy was performed in each sputum sample before culture. Samples with squamous epithelial cells > 10 per low-power field were considered unqualified and sputum culture would not be performed. Otherwise, samples were considered qualified. Each qualified specimen was inoculated onto sheep blood (Wenzhou kont biology and technology, Ltd., China), chocolate, and MacConkey agars, and incubated at 35 °C for 48 h. Cultures were examined at 24 h and 48 h, and predominant organisms were identified when there was only one type of bacteria or the quantity of one bacterial type is larger than others on semiquantitative culture.

### Statistical analysis

BALF culture was taken as the gold standard for comparison. Sensitivity was calculated as the proportion of the number of positive test results within patients with positive BALF cultures. Specificity was calculated as the proportion of the number of negative test results within patients with negative BALF cultures. A kappa value was used to assess the agreement between each test method and BALF culture. Kappa value of one showed perfect agreement and value of zero showed no agreement. Kappa value between 0.21 and 0.40 was considered as fair agreement, that between 0.41 and 0.60 was moderate and between 0.61 and 0.80 was taken as good and that showing between 0.81 and 1 was very good agreement [12]. The collected data was analyzed by SPSS version 26.0.

### Ethics approval and consent to participate

This study was performed in accordance with the Declaration of Helsinki and was approved by the ethics committee of Shenzhen Children’s Hospital with judgment’s reference number 201907903. Informed consent was obtained from patients’ legal guardians.

## Results

### Patients

**Fig. 1:**
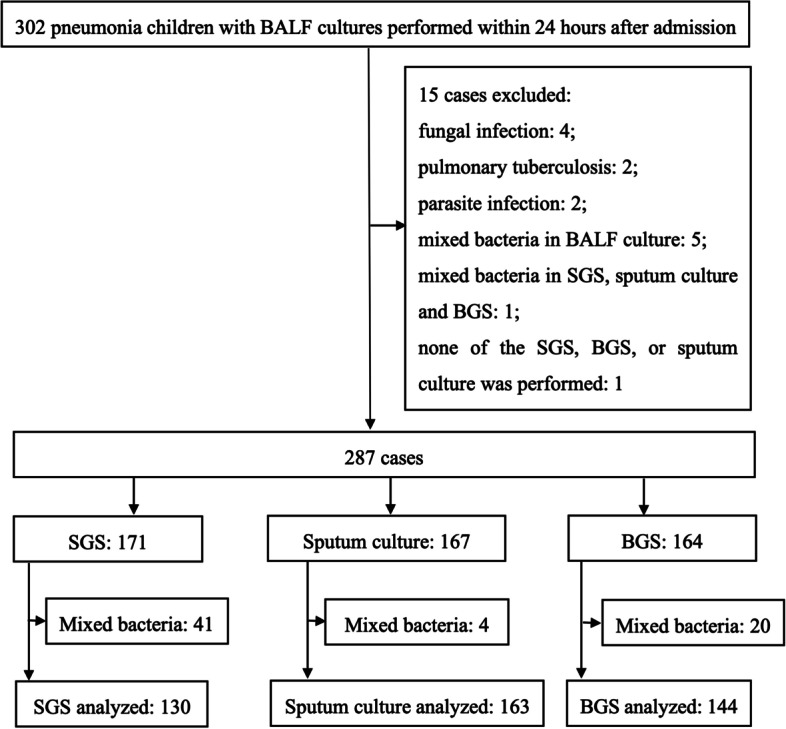
Study profile. BALF, bronchoalveolar lavage fluid; SGS, sputum Gram stain; BGS, bronchoalveolar lavage fluid Gram stain

From January 1, 2015 to December 31, 2021, 59786 children with pneumonia were admitted in our hospital and 302 received bronchoscopy within 24 h after admission. Among them, 287 children were included in the study (Fig. 1). The median age was 38 months (range: 1 month to 15 years); 166 were males and 121 were females. Underlying chronic diseases are present in 102 children, including 67 with chronic respiratory diseases, 13 with neurologic diseases, 7 with cardiovascular diseases, 5 with hematologic diseases, 3 with immune diseases, and 3 with malnutrition. Among the 185 previously healthy children, 23 developed atelectasis, and 19 developed plastic bronchitis.

### BALF culture

The positive rate of BALF culture was 34.84% (100/287). Major isolates were *Streptococcus pneumoniae, Haemophilus influenzae,* and *Moraxella catarrhalis* (Table 1). Based on the morphology, BALF isolates were classified as G+cocci, G-cocci, G+bacilli, and G-bacilli for further calculation.

**Table 1:** BALF culture results in children with pneumonia (*n* = 287)

Pathogen^a^	n (%)
*Streptococcus pneumoniae*	35(12.20)
*Haemophilus influenzae*	26(9.06)
*Moraxella catarrhalis*	18(6.27)
*Staphylococcus aureus*	8(2.79)
*Haemophilus parainfluenzae*	2(0.70)
*Klebsiella pneumoniae*	2(0.70)
*Group A streptococcus*	2(0.70)
*Pseudomonas aeruginosa*	2(0.70)
*Corynebacterium pseudodiphtheriticum*	2(0.70)
*Viridans streptococcus*	1(0.35)
*Escherichia coli*	1(0.35)
*Stenotrophomonas maltophilia*	1(0.35)
Negative	187(65.16)

*BALF* Bronchoalveolar lavage fluid

^a^ The listed pathogens are the dominant isolates from BALF cultures

### SGS

SGS smears were performed in 171 cases. The specificity of SGS was 23%. After excluding cases of mixed bacteria, the overall sensitivity of SGS was 70%, including 87% for G+cocci, 56% for G-cocci, and 50% for G-bacilli. The sputum smear and BALF culture had poor agreement (Table 2).

**Table 2:** Sensitivity, specificity, and kappa value of SGS (*n* = 171) ^a^

	BALF culture n (%)	SGS n (%)	Sensitivity %(95% CI) ^b^	Specificity %(95% CI)^b^	Kappa value (95% CI) ^b^
G+cocci	28(16.37)	74(43.27)	87(65–97)	-	-
G-cocci	9(5.26)	17(9.94)	56(23–85)	-	-
G+bacilli	0	2(1.17)	-	-	-
G-bacilli	17(9.94)	13(7.60)	50(23–78)	-	-
Mixed bacteria	0	41(23.98)	-	-	-
Negative	117(68.42)	24(14.04)	-	-	-
Total	171(100.00)	171(100.00)	70(55–83)	23(15–34)	0.199(0.107–0.292)

*SGS* Sputum Gram stain, *BALF* Bronchoalveolar lavage fluid, *CI* Confidence interval, *G*+*cocci* Gram-positive cocci, *G-cocci* Gram-negative cocci, *G*+*bacilli* Gram-positive bacilli, *G-bacilli* Gram-negative bacilli

^a^ Reference standard is bronchoalveolar lavage fluid culture

^b^ Calculation was performed after mixed bacteria cases were excluded

### Sputum culture

Sputum cultures were performed in 167 cases. The major isolates were the same as those found in BALF cultures. Mixed bacteria were found in 4 cases: 3 cases of *Haemophilus influenzae* and *Moraxella catarrhalis,* and 1 case of *Streptococcus pneumoniae* and *Moraxella catarrhalis.* The specificity was 70%. The overall sensitivity was 64%, including 71% for *Streptococcus pneumoniae*, 71% for *Moraxella catarrhalis*, and 64% for *Haemophilus influenzae*. The sputum culture and BALF culture had moderate agreement (Table 3).

**Table 3:** Sensitivity, specificity, and kappa value of sputum culture (*n* = 167) ^a^

	BALF culture n (%)	Sputum culture n (%)	Sensitivity %(95% CI) ^b^	Specificity %(95% CI)^b^	Kappa value (95% CI) ^b^
*Streptococcus pneumoniae*	21(12.57)	24(14.37)	71(48–88)	-	-
*Haemophilus influenzae*	12(7.19)	11(6.59)	64(32–88)	-	-
*Moraxella catarrhalis*	7(4.19)	22(13.17)	71(30–95)	-	-
Mixed bacteria	0	4(2.40)	-	-	-
Others	14(8.38)	12(7.19)	-	-	-
Negative	113(67.66)	94(56.29)	-	-	-
Total	167(100.00)	167(100.00)	64(50–77)	70(60–78)	0.450(0.328–0.571)

*BALF* Bronchoalveolar lavage fluid, *CI* Confidence interval

^a^ Reference standard is bronchoalveolar lavage fluid culture

^b^ Calculation was performed after mixed bacteria cases were excluded

### BGS

BGS smears were performed in 164 cases. The specificity of BGS was 71%. After excluding cases of mixed bacteria, the overall sensitivity of BGS was 60%, including 77% for G+cocci, 38% for G-cocci, and 44% for G-bacilli. The BGS and BALF culture had moderate agreement (Table 4).

**Table 4:** Sensitivity, specificity, and kappa value of BGS (*n* = 164) ^a^

	BALF culture n (%)	BGS n (%)	Sensitivity %(95% CI) ^b^	Specificity %(95% CI)^b^	Kappa value (95% CI) ^b^
G+cocci	29(17.68)	48(29.27)	77(56–90)	-	-
G-cocci	10(6.10)	5(3.05)	38(10–74)	-	-
G+bacilli	0	1(0.61)	-	-	-
G-bacilli	22(13.41)	14(8.54)	44(22–69)	-	-
Negative	103(62.80)	76(46.34)	-	-	-
Mixed bacteria	0	20(12.20)	-	-	-
Total	164(100.00)	164(100.00)	60(45–73)	71(60–79)	0.434(0.313–0.555)

*BGS* bronchoalveolar lavage fluid Gram stain, *BALF* bronchoalveolar lavage fluid, *CI* Confidence interval, *G*+*cocci* Gram-positive cocci, *G-cocci* Gram-negative cocci, *G*+*bacilli* Gram-positive bacilli, *G-bacilli* Gram-negative bacilli

^a^ Reference standard is bronchoalveolar lavage fluid culture

^b^ Calculation was performed after mixed bacteria cases were excluded

## Discussion

In medical practice, an early identification of the pathogen is important for the treatment of bacterial pneumonia [13]. Even simple clues for the bacterial type are helpful to early clinical decisions, as most antibiotics target a group of bacteria, not a specific bacterium. Though polymerase chain reaction assays identify specific bacteria in hours, it can only detect targeted bacteria, which may miss some atypical bacteria or normal flora causing pneumonia, and has relatively high lab requirements. Culture provides more accurate evidence of bacterial infection, but it has limited sensitivity and long turnaround time [14].

The accuracy of SGS is in doubt, though it is quick and convenient in use. Previous studies of SGS suggested it was sensitive and specific for etiologic pathogens of bacterial pneumonia, when sputum culture was taken as the reference standard [8, 15, 16]. However, it is controversial to make etiologic diagnosis in pneumonia by sputum culture results, due to concerns of representativeness and contamination [4]. BALF is commonly accepted as the ideal specimen for causative pathogen detection. The value of SGS would be more accurately assessed when BALF culture is used as the reference standard.

In our study, the overall sensitivity of SGS was 70%, with variety in different bacterial types. By contrast, the specificity and the agreement with BALF culture were both poor, indicating the limited value of SGS in predicting BALF pathogens. Previous studies suggested the specificity of SGS for G-cocci could be up to 98%, as it was calculated as the proportion of negative G-cocci SGSs among negative G-cocci cultures [16]. In that way, the concordance between SGS and sputum culture was neglected. For example, the SGS could be G+cocci and the culture could be G-bacilli, though they are both negative for G-cocci. In our study, specificity was only calculated as the proportion of negative SGSs among negative BALF cultures, revealing the poor specificity of SGS.

One obvious disadvantage of sputum culture is the difficulty to attribute causality. Data on its concordance with BALF culture is helpful in answering this question. Of note, the interval between sputum culture and BALF culture was seldom mentioned in previous studies. Comparison of these two methods may be biased when the interval is long. This was avoided to a great extent in our study, as the interval was less than 24 h. In our study, the agreement between sputum and BALF, rather than a simple comparison of pathogen positive rates, was analyzed. The moderate agreement suggested sputum culture is helpful in predicting BALF culture.

It is unclear whether BGS can provide clues for etiologic pathogens of pneumonia. Theoretically, it is valuable as BALF is an ideal specimen type and Gram stain is quick. In our study, its sensitivity was 60% and specificity was 71%. There was a moderate agreement between BGS and BALF culture, supporting its value in etiologic diagnosis when BALF culture result is pending.

This study has limitations due to the retrospective nature and small sample size. A prospective study with simultaneous collection of sputum and BALF specimens can avoid the time interval of different specimens. As expectorated sputum and nasotracheal suctioned sputum samples were both named “sputum” in the lab report, the specific sputum origin was not clear. Sample sizes of SGS, sputum culture, and BGS would be greatly increased if these tests were performed in every case. Data would be richer if polymerase chain reaction assays or whole-genome sequencing for bacteria were performed [17–19].

## Conclusion

Both sputum culture and BGS are helpful in predicting single bacterial pathogen in BALF among children with community-acquired pneumonia. Sputum culture and BGS can provide early clues for BALF pathogens when BALF culture results are pending or bronchoscopy is not performed.

## Data Availability

The datasets used and/or analyzed during the current study are available from the corresponding author on reasonable request.

## Abbreviations

BALF: Bronchoalveolar lavage fluid
SGS: Sputum Gram stain
BGS: Bronchoalveolar lavage fluid Gram stain
G+: Gram-positive
G-: Gram-negative
